# Validation of commercially available sphingosine kinase 2 antibodies for use in immunoblotting, immunoprecipitation and immunofluorescence

**DOI:** 10.12688/f1000research.10336.2

**Published:** 2017-03-23

**Authors:** Heidi A. Neubauer, Stuart M. Pitson

**Affiliations:** 1 Centre for Cancer Biology, University of South Australia and SA Pathology, Adelaide, Australia; 2 School of Biological Sciences, University of Adelaide, Adelaide, Australia; 3 School of Medicine, University of Adelaide, Adelaide, Australia

**Keywords:** Sphingosine kinase 2; antibody validation; immunoblotting, immunoprecipitation, immunofluorescence

## Abstract

Sphingosine kinase 2 (SK2) is a ubiquitously expressed lipid kinase that has important, albeit complex and poorly understood, roles in regulating cell survival and cell death. In addition to being able to promote cell cycle arrest and apoptosis under certain conditions, it has recently been shown that SK2 can promote neoplastic transformation and tumorigenesis *in vivo*. Therefore, well validated and reliable tools are required to study and better understand the true functions of SK2. Here, we compare two commercially available SK2 antibodies: a rabbit polyclonal antibody from Proteintech that recognizes amino acids 266-618 of human SK2a, and a rabbit polyclonal antibody from ECM Biosciences that recognizes amino acids 36-52 of human SK2a. We examine the performance of these antibodies for use in immunoblotting, immunoprecipitation and immunofluorescence staining of endogenous SK2, using human HEK293 and HeLa cell lines, as well as mouse embryonic fibroblasts (MEFs). Furthermore, we assess the specificity of these antibodies to the target protein through the use of siRNA-mediated SK2 knockdown and SK2 knockout ( *Sphk2* ^-/-^) MEFs. Our results demonstrate that the Proteintech anti-SK2 antibody reproducibly displayed superior sensitivity and selectivity towards SK2 in immunoblot analyses, while the ECM Biosciences anti-SK2 antibody was reproducibly superior for SK2 immunoprecipitation and detection by immunofluorescence staining. Notably, both antibodies produced non-specific bands and staining in the MEFs, which was not observed with the human cell lines. Therefore, we conclude that the Proteintech SK2 antibody is a valuable reagent for use in immunoblot analyses, and the ECM Biosciences SK2 antibody is a useful tool for SK2 immunoprecipitation and immunofluorescence staining, at least in the human cell lines employed in this study.

## Introduction

Sphingolipids are an important family of cellular molecules that form critical structural components of cell membranes, as well as performing numerous signaling functions ^1^. Of the many enzymes responsible for the biosynthesis and metabolism of sphingolipids, the sphingosine kinases (SKs) are of particular interest to study as they catalyze the formation of sphingosine 1-phosphate (S1P), and in doing so can promote cell survival, proliferation, migration and angiogenesis ^2^. Both sphingosine kinases, SK1 and SK2, have been shown to be upregulated in various human cancers and both have documented roles in mediating oncogenesis ^3, 4^. However, where SK1 and its roles in cancer development are relatively well characterized, SK2 remains somewhat enigmatic as, in addition to the pro-cancer functions it shares with SK1, SK2 can also facilitate cell cycle arrest and cell death ^5, 6^.

SK2 is ubiquitously expressed in all cells and tissues, but is expressed most highly in the liver, kidney and brain ^7^. At the mitochondria, SK2-generated S1P has been proposed to facilitate the activation of Bak and subsequent mitochondrial membrane permeabilisation and cytochrome c release ^5^. Notably, SK2 can also function as an epigenetic regulator, where S1P produced by nuclear-localized SK2 can inhibit the activity of histone deacetylases 1/2 resulting in increased transcription of specific genes, such as cyclin-dependent kinase inhibitor *p21* and transcriptional regulator *c-fos* ^8^. As SK1 does not appear to localize as prominently to these internal organelles, it is believed that the subcellular localization of SK2 is critical for the additional functions it performs. However, the mechanisms regulating the localization and functions of SK2, allowing it to switch between pro-apoptotic and pro-survival under certain conditions, remain poorly understood.

In order to study SK2 and better characterize its roles in normal cells as well as in cancer, reliable and properly validated tools are required. Antibody-based methods, such as immunoblotting (IB), immunoprecipitation (IP) and immunofluorescence (IF), are particularly useful as tools to examine and visualize important aspects of SK2 biology, like subcellular localization, expression and interaction with regulatory proteins. A number of groups in the field have taken to generating their own in-house SK2-specific polyclonal antibodies ^9, 10^, but to our knowledge there has been no systematic validation of commercially available SK2 antibodies. Here, we compare two commercially available SK2 antibodies, and validate the suitability of their use in IB, IP and IF using various human and mouse cells lines. We have examined a rabbit polyclonal SK2 antibody from Proteintech, which is raised against amino acids 266–618 of recombinant human SK2a, and a rabbit polyclonal SK2 antibody from ECM Biosciences, which is raised against a synthetic peptide corresponding to amino acids 36–52 of human SK2a. The Proteintech SK2 antibody has been previously utilized in one publication for IB ^11^, and the ECM Biosciences SK2 antibody has been used in multiple publications for IB ^12– 15^ and for IF ^16^.

## Materials and methods

### Antibody details

The following SK2 antibodies were assessed: rabbit polyclonal anti-SK2 (ECM Biosciences; anti-Sphingosine Kinase 2 (N-terminal region); #SP4621, lot #1) and rabbit polyclonal anti-SK2 (Proteintech Group, Inc; anti-SPHK2; #17096-1-AP, lot #00010361). The ECM Biosciences SK2 antibody was raised against a synthetic peptide coupled to keyhole limpet hemocyanin (KLH), corresponding to amino acids 36–52 of human SK2a, and was affinity purified with the SK2 peptide (without KLH). It is reported by the manufacturer to have cross-reactivity with rat and mouse SK2 [human, mouse and rat SK2 share 100% sequence identity in this region (determined using the align tool and protein sequences from www.uniprot.org)], and has been assessed by the manufacturer for use in IB and enzyme-linked immunosorbent assay (ELISA). The Proteintech SK2 antibody was raised against truncated recombinant GST-tagged human SK2a (amino acid residues 266–618 generated in *Escherichia coli* using the PGEX-4T plasmid). The SK2 target antibodies were then affinity purified using 6xHis-tagged antigen protein (to remove GST-specific antibodies) and then again with the immunising GST-tagged antigen protein. It is reported to have cross-reactivity with rat and mouse SK2 [80.2% sequence identity between human and mouse SK2, and 80.2% sequence identity between human and rat SK2 in this region (determined using the align tool and protein sequences from www.uniprot.org)], and according to the manufacturer can be employed for IB, ELISA, IP and immunohistochemistry. Mouse anti-α-tubulin (DM1A; Abcam; #ab7291) is a mouse monoclonal antibody, which was used as a loading control for IB analyses, at a dilution of 1:5,000. All antibody details, including information for secondary antibodies used, are provided in Table 1.

**Table 1.** Details of primary and secondary antibodies.

Antibody	Manufacturer	Catalogue number	RRID
Rabbit anti-SK2	ECM Biosciences	SP4621	AB_2619719
Rabbit anti-SK2	Proteintech	17096-1-AP	AB_10598479
Mouse anti-α-tubulin	Abcam	ab7291	AB_2241126
Goat anti-rabbit IgG HRP	Thermo Fisher Scientific	31460	AB_228341
Goat anti-mouse IgG HRP	Thermo Fisher Scientific	31430	AB_228307
Normal rabbit IgG	Cell Signaling Technology	2729	AB_2617119
Goat anti-rabbit Alexa Fluor 488	Thermo Fisher Scientific	A-11008	AB_143165

### Cell culture

Human embryonic kidney (HEK) 293 cells (CellBank Australia; #85120602) and HeLa human cervical cancer cells (ATCC; #CCL-2) were cultured in Dulbecco’s modified Eagle’s medium (DMEM; Gibco, Thermo Fisher Scientific Inc.), containing 10% heat-inactivated fetal bovine serum (FBS; Bovagen), 1 mM HEPES, penicillin (1.2 mg/ml) and streptomycin (1.6 mg/ml). Cells were grown at 37°C with 5% CO _2_ in a humidified incubator. Primary mouse embryonic fibroblasts (MEFs) were generated from both wildtype (WT) C57/Bl6 and *Sphk2* ^-/-^ C57/Bl6 ^17^ mouse embryos at 14.5 days *post coitum*. The fibroblasts were isolated and cultured as described above, except they were maintained at 37°C in a humidified atmosphere with 10% CO _2_.

### RNAi knockdown of SK2

siRNA-mediated knockdown of SK2 was performed using human SPHK2 siGENOME SMARTpool siRNA (Dharmacon), which targets the following sequences: CCACUGCCCUCACCUGUCU, GCUCCUCCAUGGCGAGUUU, GAGACGGGCUGCUCCAUGA, CAAGGCAGCUCUACACUCA. Cells were seeded and grown to a cell density of approximately 50%, and were then transfected with 30 nM (final concentration) of either human SK2 siRNA or siGENOME non-targeting siRNA control pool (Dharmacon), using Lipofectamine RNAiMAX (Life Technologies), as per the manufacturer’s protocol. Cells were incubated with the siRNA complexes at 37°C for 48 h.

### Immunoblotting

Specific details for all reagents used can be found in Table 2. Cells were pelleted by centrifugation (400 × *g*, 5 min, 4°C) and washed in cold phosphate buffered saline (PBS). Cell pellets were resuspended in extraction buffer [EB; 50 mM Tris/HCl buffer (pH 7.4) containing 150 mM NaCl, 10% glycerol, 1 mM EDTA, 0.05% Triton X-100, 2 mM Na _3_VO _4_, 10 mM NaF, 10 mM β-glycerophosphate, 1 mM dithiothreitol (DTT) and protease inhibitor cocktail (Roche)], and lysed by bath sonication (4× 30 sec on/off). Lysates were clarified (17,000 × *g*, 15 min, 4°C) and equal amounts of protein [as determined by a Bradford protein assay (Bio-Rad Laboratories)] were mixed with 5× Laemmli sample buffer, boiled at 100°C for 5 min, and separated by SDS-PAGE on a Criterion™ XT Bis-Tris 4–12% gradient gel (Bio-Rad Laboratories). Proteins were then transferred to nitrocellulose membrane (Pall Life Sciences) at 400 mA for 1 h. Membranes were blocked with 5% skim milk in PBS containing 0.1% Triton X-100 (PBS-T) for 1 h at room temperature, with gentle rocking. Membranes were probed with rabbit anti-SK2 antibodies diluted in Signal Boost primary antibody diluent at 1:1,000 (ECM Biosciences: 1 µg/ml; Proteintech: 687 ng/ml) overnight at 4°C with gentle rocking. Alternatively, membranes were probed with mouse anti-α-tubulin antibody diluted in 5% skim milk in PBS-T at 1:5,000 (200 ng/ml) for 1 h at room temperature, with gentle rocking. Following primary antibody incubation, membranes were washed 3 × 5 min in 5% skim milk in PBS-T at room temperature with gentle agitation. Membranes were probed with goat anti-rabbit horseradish peroxidase (HRP) secondary antibody diluted in Signal Boost secondary antibody diluent at 1:10,000 (40 ng/ml), or goat anti-mouse HRP secondary antibody diluted in 5% skim milk in PBS-T at 1:10,000 (40 ng/ml), for 1 h at room temperature, with gentle rocking. Membranes were washed 3 × 5 min in 5% skim milk in PBS-T at room temperature with gentle agitation, and proteins were visualized using enhanced chemiluminescence (ECL) on a LAS-4000 luminescence image analyser (Fujifilm). Exposure times are indicated in the figure legends for each blot.

**Table 2.** Reagents used for immunoblotting.

Process	Reagent	Manufacturer	Catalogue number	Concentration
Protein Concentration Assay	Protein Assay Dye Reagent Concentrate	Bio-Rad Laboratories	500-0006	Proprietary
5x Laemmli Protein Loading Buffer	Tris/HCl pH 8.0 EDTA Dithiothreitol (DTT) SDS Glycerol Bromophenol blue	Invitrogen Merck Millipore Sigma Aldrich Sigma Aldrich Chem-Supply Sigma Aldrich	15504-020 108421 D0632 75746 GA010 B5525	50 mM 5 mM 100 mM 5% w/v 50% v/v 0.1% w/v
SDS-PAGE Running Buffer 1×	Tris/HCl pH 7.3 MES SDS EDTA	Invitrogen Amresco Sigma Aldrich Merck Millipore	15504-020 E169 75746 108421	50 mM 50 mM 0.1% w/v 1 mM
SDS-PAGE Transfer Buffer 1×	Tris Glycine Methanol	Invitrogen MP Biomedicals RCI Labscan Ltd	15504-020 04808831 AR1115	25 mM 192 mM 20% v/v
Blocking Buffer, Antibody Diluent, Wash Buffer	Skim milk powder PBS-T (PBS, Triton X-100)	Diploma Merck Millipore	108643	5% w/v 0.1% v/v
Antibody Diluent	SignalBoost Immunoreaction Enhancer Kit	Calbiochem	407207	Proprietary
Enhanced Chemiluminescence	Clarity Western ECL Substrate	Bio-Rad Laboratories	170-5061	Proprietary

### Immunoprecipitation

HEK293 cell lysates were prepared as for immunoblotting, with the exclusion of DTT in the extraction buffer (EB–DTT). Protein concentration was determined from clarified lysates, and 800 µg total protein was transferred to fresh tubes and made up to 300 µl in EB–DTT. In total, 20 µl of diluted lysate was removed and mixed with 5× Laemmli sample buffer for immunoblot analysis. Immunoprecipitation was performed using the µMacs magnetic system (Miltenyi Biotec; see Table 3 for reagent details). Rabbit anti-SK2 antibodies (4 µg; ECM Biosciences 1:75 or Proteintech 1:52) or rabbit IgG isotype control antibody (4 µg), as well as 50 µl each of Protein A and G µBeads were added to the lysate, mixed gently and incubated on ice for 30 min. µMacs columns were placed onto a magnetic stand, equilibrated with 200 µl EB–DTT, and lysate/antibody/bead complexes were run through the columns. Columns were washed four times with 200 µl EB–DTT, and once with 100 µl low salt wash buffer, before the addition of 20 µl hot 1× Laemmli sample buffer for 5 min. Immunoprecipitates were then eluted with 50 µl hot 1× Laemmli sample buffer and collected in fresh tubes. Samples were boiled and 25 µl was analysed by SDS-PAGE and immunoblotting as described above.

**Table 3.** Reagents used for immunoprecipitation

Process	Reagent	Manufacturer	Catalogue number	Concentration
Magnetic Labelling (µMacs)	Protein A µBeads Protein G µBeads	Miltenyi Biotech	130-071-001 130-071-101	Proprietary
Washing Columns	EB -DTT (described in methods) Low Salt Wash Buffer: Tris/HCl pH 7.5	Invitrogen	15504-020	Various 20 mM
1× Laemmli Protein Loading Buffer	Tris/HCl pH 8.0 EDTA Dithiothreitol (DTT) SDS Glycerol Bromophenol blue	Invitrogen Merck Millipore Sigma Aldrich Sigma Aldrich Chem-Supply Sigma Aldrich	15504-020 108421 D0632 75746 GA010 B5525	10 mM 1 mM 20 mM 1% w/v 10% v/v 0.02% w/v

### Immunofluorescence staining

Cell lines were seeded onto coverslips coated with poly-L-lysine (Sigma-Aldrich) in a 12-well plate (HEK293: 1.5 ×10 ^5^ cells/well; HeLa: 7.5 ×10 ^4^ cells/well; MEF: 3 ×10 ^4^ cells/well). Cells were allowed to bed down overnight at 37°C in DMEM with 10% FBS. The following day (for HEK293 and HeLa cells), cells were treated with siRNA as described above, and incubated for 48 h. The following immunofluorescence staining protocol was then performed, with all steps carried out at room temperature (see Table 4 for reagent details). Cells were washed once in PBS, fixed in 4% paraformaldehyde for 10 min, washed three times in PBS-T, and permeabilized for 10 min in PBS-T. Cells were then blocked in 3% bovine serum albumin (BSA) in PBS-T for 30 min, and incubated for 1 h with anti-SK2 antibodies (4 µg/ml; ECM Biosciences 1:250 and Proteintech 1:172) diluted in 3% BSA/PBS-T. Cells were washed five times in PBS-T, and then incubated with goat anti-rabbit AlexaFluor 488 secondary antibody (1:500) for 1 h. After washing five times in PBS-T, cell nuclei were stained with DAPI (0.2 µg/ml) for 5 min. Cells were then washed twice in PBS, coverslips were partially dried and mounted onto slides using fluorescence mounting medium (Dako), and then left to set overnight. Fluorescence microscopy and imaging were performed using a Carl Zeiss LSM 700 confocal microscope, with Zen 2011 (Black Edition) version 8.1.5.484 software. All microscope settings, including gains, were kept constant for each cell line, allowing direct comparison between antibodies.

**Table 4.** Reagents used for immunofluorescence

Process	Reagent	Manufacturer	Catalogue number	Concentration
Washing	PBS			1x
Fixing	Paraformaldehyde	Santa Cruz Biotechnology	sc-281692	4% in PBS
Permeabilizing, Washing	PBS-T			0.1% Triton X-100 in PBS
Blocking, Antibody Diluent	Bovine Serum Albumin (BSA) PBS-T	Sigma Aldrich	A7906	3% w/v
Nuclear Staining	DAPI (4',6-diamidino-2- phenylindole)	Roche	10236276001	0.2 µg/ml in PBS
Mounting	Fluorescent Mounting Medium	Dako	S3023	Proprietary

### Controls

Various controls were used in these studies. Immunoprecipitations were performed with IgG isotype control antibody to control for any non-specific binding of proteins to the antibodies. Primary antibodies were also omitted from the immunofluorescence protocol to control for background fluorescence of the secondary antibody alone. siRNA-mediated SK2 knockdown as well as *Sphk2* ^-/-^ MEFs were utilized to verify the specificity of the SK2 antibodies to their target.

## Results

### Proteintech SK2 antibody demonstrates target specificity and sensitivity by immunoblot

**Figure 1.**
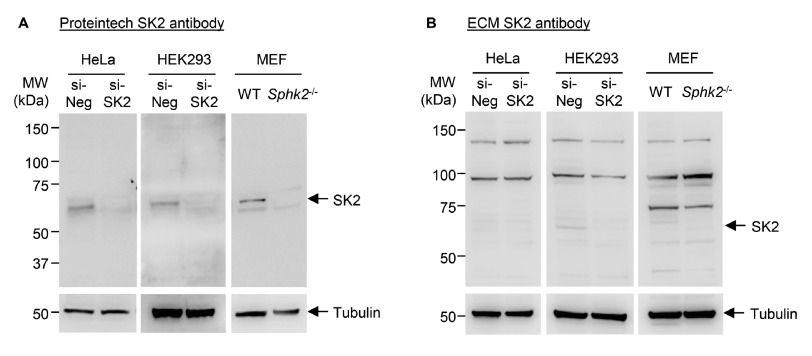
Immunoblot analyses of endogenous SK2 in multiple cell lines using two commercially available rabbit polyclonal anti-SK2 antibodies. Immunoblot analyses of lysates from HEK293 and HeLa cells treated with scrambled control siRNA (si-Neg) or SK2 siRNA (si-SK2), and lysates from wildtype (WT) or *Sphk2* ^-/-^ MEFs. An equal amount (40 µg) of total protein from each sample was run in duplicate. After transferring to nitrocellulose and blocking, the membrane was separated and duplicate samples were probed with either ( **A**) Proteintech rabbit anti-SK2 antibody or ( **B**) ECM Biosciences rabbit anti-SK2 antibody. SK2 membranes were imaged using a 4 min exposure. The expected band size for SK2 is ∼65 kDa. Membranes were re-probed with mouse anti-α-tubulin antibody as a loading control (2 min exposure), which was detected at 55 kDa as expected. Consistent results were observed from 2-3 (HEK293 and MEF) or 3-4 (HeLa) independent experiments for each antibody.

Both SK2 antibodies examined in the present study are reported by their respective manufacturers to be able to detect endogenous SK2 by IB. To determine the selectivity of the anti-SK2 antibodies, we performed IB analyses using two human cell lines (HEK293 and HeLa) that had been treated with either scrambled control or SK2-directed siRNA, as well as WT and *Sphk2* ^-/-^ MEFs. The Proteintech anti-SK2 antibody detected a single prominent band at the correct molecular weight for SK2 (~65 kDa), which was decreased or absent in the knockdown and knockout lines ( Figure 1A; Dataset 1 ^18^). Some faint non-specific bands were also detected in both the WT and *Sphk2* ^-/-^ MEF lysates by this antibody, which were not observed in the human cell lines. The ECM Biosciences anti-SK2 antibody did not appear to be very sensitive towards SK2, as no band was detected at the expected size in the HeLa lysates, and only very faint bands were present in the HEK293 and MEF lysates that were reduced or absent in the knockdown or knockout lines ( Figure 1B). Furthermore, numerous prominent non-specific bands were present in all lysates, particularly in the MEF lines, indicating a lack of selectivity of this antibody towards SK2. Therefore, the Proteintech anti-SK2 antibody appears to be superior for use in IB, demonstrating both selectivity and sensitivity in the detection of endogenous SK2, particularly in the human cell lines tested.

Raw images of all experimental replicates for Figure 1, immunoblotting experiments Click here for additional data file. Copyright: © 2017 Neubauer HA and Pitson SM 2017 Data associated with the article are available under the terms of the Creative Commons Zero "No rights reserved" data waiver (CC0 1.0 Public domain dedication).

### ECM Biosciences SK2 antibody is able to specifically immunoprecipitate SK2

**Figure 2.**
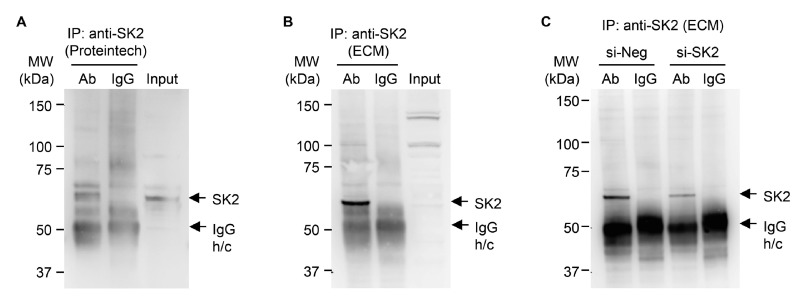
Comparison of two commercially available rabbit polyclonal anti-SK2 antibodies for immunoprecipitation of SK2 from HEK293 cell lysates. SK2 was immunoprecipitated from HEK293 cell lysate using either ( **A**) Proteintech rabbit anti-SK2 antibody or ( **B**) ECM Biosciences rabbit anti-SK2 antibody. Normal rabbit IgG antibody was used as an isotype control. Immunoprecipitates (and 40 µg lysate input) were subjected to immunoblot analyses and probed with ( **A**) Proteintech rabbit anti-SK2 antibody or ( **B**) ECM Biosciences rabbit anti-SK2 antibody. Membranes were imaged using a 4 min exposure. Images are representative of three independent experiments for each antibody. ( **C**) SK2 was immunoprecipitated from HEK293 cell lysates (of equal protein) that had been treated with scrambled control siRNA (si-Neg) or SK2 siRNA (si-SK2), using ECM Biosciences rabbit anti-SK2 antibody. Immunoprecipitates were subjected to immunoblot analyses and probed with ECM Biosciences rabbit anti-SK2 antibody. Membrane was imaged using a 4 min exposure. Image is representative of three independent experiments. IgG h/c = IgG heavy chain.

We also examined whether either of the commercial anti-SK2 antibodies could immunoprecipitate SK2 from cell lysates. The Proteintech anti-SK2 antibody is suggested by the manufacturer to be useful for IP, whereas to our knowledge the ECM Biosciences anti-SK2 antibody has not been previously tested for use in this application. Initially, using lysates from HEK293 cells, we found that the Proteintech anti-SK2 antibody was sometimes able to IP a band at the correct size for SK2 ( Figure 2A); however this was not consistent with each experimental repeat and other proteins were also immunoprecipitated to a varying extent by this antibody that were not present in the IgG isotype control ( Dataset 2 ^19^).

Conversely, the ECM Biosciences anti-SK2 antibody was able to consistently and cleanly IP a protein of the same size as SK2 from cell lysates, with almost no non-specific bands observed ( Figure 2B; Dataset 2 ^19^). The protein immunoprecipitated by the ECM Biosciences antibody was considerably enriched from the cell lysate and was strongly detectable by this antibody, which was unable to detect SK2 in the lysate input sample, consistent with Figure 1B. To determine if this band was in fact SK2, the ECM Biosciences anti-SK2 antibody was then used to immunoprecipitate SK2 from HEK293 lysates treated with either scrambled control or SK2-directed siRNA. SK2 knockdown consistently resulted in reduced intensity of the band enriched by this antibody ( Figure 2C; Dataset 2 ^19^), confirming that the ECM Biosciences anti-SK2 antibody can selectively IP endogenous SK2.

Raw images of all experimental replicates for Figure 2, immunoprecipitation experiments Click here for additional data file. Copyright: © 2017 Neubauer HA and Pitson SM 2017 Data associated with the article are available under the terms of the Creative Commons Zero "No rights reserved" data waiver (CC0 1.0 Public domain dedication).

### ECM Biosciences SK2 antibody can specifically detect SK2 by immunofluorescence staining

**Figure 3.**
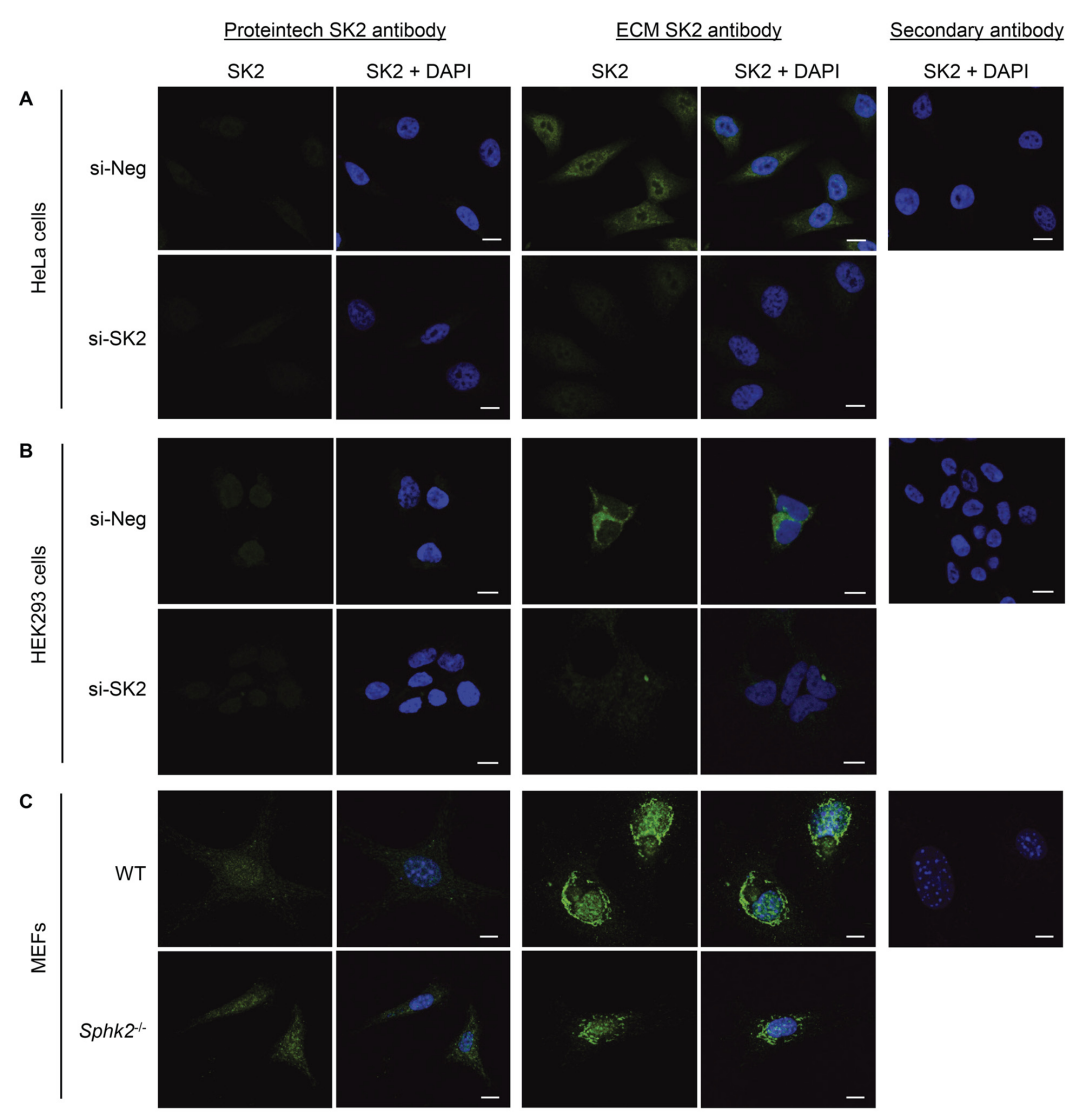
Immunofluorescence staining analyses of endogenous SK2 in multiple cell lines using two commercially available rabbit polyclonal anti-SK2 antibodies. ( **A**) HeLa or ( **B**) HEK293 cells were treated with scrambled control siRNA (si-Neg) or SK2 siRNA (si-SK2), and endogenous SK2 (green) was visualised by immunofluorescence staining and confocal microscopy, using Proteintech rabbit anti-SK2 antibody or ECM Biosciences rabbit anti-SK2 antibody. ( **C**) Wildtype (WT) or *Sphk2* ^-/-^ MEFs were seeded, and endogenous SK2 (green) was visualised by immunofluorescence staining and confocal microscopy, using Proteintech rabbit anti-SK2 antibody or ECM Biosciences rabbit anti-SK2 antibody. Nuclei were stained with DAPI (blue). For each cell line, background staining was examined by staining cells (si-Neg or WT cells) with secondary antibody and DAPI only, and collecting images using both 488nm and 405nm lasers (SK2 + DAPI). Images were taken at 40× magnification; scale bars = 10 µm. Images shown are representative of more than 100 cells from each experiment, and these results were consistent over three independent experiments for each cell line.

Finally, we examined whether these commercially available SK2 antibodies could selectively detect SK2 by IF. Neither antibody has been reported to be tested for use in IF by their respective manufacturers; however, the Proteintech SK2 antibody is recommended for immunohistochemistry. Using IF staining methods routinely performed in our laboratory, we compared the two anti-SK2 antibodies using HeLa, HEK293 and MEF cell lines. The Proteintech anti-SK2 antibody produced minimal staining in all cell lines tested ( Figure 3A–C), and consequently there was no observable differences between the control cells and those with SK2 knockdown (in the human cell lines) or SK2 knockout (in the *Sphk2* ^-/-^ MEF line).

The ECM Biosciences anti-SK2 antibody did result in consistently observable staining in HeLa and HEK293 cells, which was substantially reduced upon knockdown of SK2 ( Figure 3A and B; Dataset 3 ^20^). Hence, in these cells the ECM Biosciences antibody was able to selectively detect SK2 by IF. Interestingly, in HeLa cells SK2 detected by the ECM Biosciences antibody was predominantly nuclear with some peri-nuclear/cytoplasmic localization, whereas in HEK293 cells SK2 was cytoplasmic and nuclear-exclusion, which is consistent with previous reports for these cell lines ^9^. However, the ECM Biosciences anti-SK2 antibody produced very strong peri-nuclear staining/puncta in both the WT and *Sphk2* ^-/-^ MEF lines ( Figure 3C), suggesting that this staining was not specific for SK2 and represents non-specific binding to other proteins in this cell type. Increased non-specific binding of both SK2 antibodies to other proteins in the MEF lines was also observed when used for IB, so this cell type may not be suitable for use with these antibodies. It will remain to be determined if the same level of non-specificity is also observed in other mouse cell lines and tissues.

Raw images of additional experimental replicates for Figure 3, immunofluorescence experiments Click here for additional data file. Copyright: © 2017 Neubauer HA and Pitson SM 2017 Data associated with the article are available under the terms of the Creative Commons Zero "No rights reserved" data waiver (CC0 1.0 Public domain dedication).

## Conclusion

Commercially available antibodies raised against the SKs can be notorious, in our experience, for not being very sensitive or selective. This is accentuated by the apparent low abundance of SK proteins in most cells, which can be in some cases hundreds of fold lower than when overexpressed ^4, 21^. A number of groups have generated their own SK-specific antibodies; however, many published studies have reported the use of different commercial SK2 antibodies, sometimes without proper controls or validation of selectivity. Hence, we have compared two commercially available SK2 antibodies and evaluated their selectivity towards SK2 in multiple applications using siRNA-mediated SK2 knockdown or *Sphk2* ^-/-^ MEF lines.

We found that the SK2 antibody from Proteintech was able to consistently detect a prominent band at the correct molecular weight by IB, and this band was confirmed to be SK2 by knockdown and knockout analyses, confirming the specificity of this antibody. The Proteintech antibody also resulted in virtually no non-specific detection of any other proteins in the HEK293 and HeLa lysates, but some additional faint bands were present in the MEF lines. This antibody has been tested by IB on various mouse tissue lysates by the manufacturer and many of these also gave rise to non-specific bands, so this will need to be considered and further validation may be required if this antibody is intended for use with mouse cells or tissues. Occasionally more than one band was detected in the human cell lines by the Proteintech SK2 antibody, but these bands also seemed to be reduced by SK2 knockdown. There are two characterized human SK2 isoforms ^22^, so these bands may represent different SK2 variants and/or post-translationally modified forms of SK2. Notably, although a previous report suggested that the mRNA of the 69 kDa SK2b (SK2-L) isoform is the most abundant in many human cell lines, including HeLa cells ^6^, our studies clearly suggest that the SK2a (SK2-S) isoform is the predominant form of SK2 protein in HeLa and HEK293 cells.

In contrast, the present results revealed that the sensitivity of the ECM Biosciences antibody towards SK2 by IB was poor, with a faint band detected only in the HEK293 and MEF lines that was not present in the knockdown/knockout lysates. Furthermore, the ECM Biosciences SK2 antibody produced many intense non-specific bands in all cell lines tested, demonstrating poor selectivity. This antibody has been used for IB analyses in multiple publications ^12– 15^, suggesting that it may be more suitable with other cell/tissue systems or conditions not tested here. However, in agreement with our findings, the IB analysis performed by the manufacturer also showed various prominent non-specific bands in HeLa lysates. Therefore, at least in our hands, the ECM Biosciences SK2 antibody was not ideal for this application.

However, the ECM Biosciences anti-SK2 antibody was superior for the IP of endogenous SK2, as it was able to cleanly and substantially enrich the protein from lysates and was confirmed by SK2-specific knockdown to be selective for SK2 in this application. This antibody will therefore be a useful tool to study SK2 function and regulation, as it can be applied to other applications requiring IP, such as chromatin-IP (ChIP) and rapid immunoprecipitation mass spectrometry of endogenous protein (RIME). In the present study, the Proteintech anti-SK2 antibody was inconsistent in its ability to IP protein at the correct size for SK2, and other bands of equal intensity were sometimes present.

Similarly, we found that the ECM Biosciences anti-SK2 antibody was able to selectively detect endogenous SK2 by IF staining in two human cell lines, HeLa and HEK293 cells. Furthermore, the observed localization of SK2 in these two cells lines was consistent with previous reports ^9^. The selectivity of this antibody was validated by knockdown of SK2 in these cell lines, where most of the staining was reduced. A very small level of staining was still visible after SK2 siRNA treatment, possibly owing to the inherently incomplete nature of siRNA-mediated knockdown. However, we were unable to corroborate these data with SK2 knockout using the MEF lines, as considerable non-specific staining was present in this cell type, as was found for IB. Using identical methods, there was minimal staining observed using the Proteintech anti-SK2 antibody for IF, and therefore the selectivity of this antibody towards SK2 in this application could not be properly examined.

During this study, methods routinely used in our laboratory were employed, and where applicable, recommendations from the manufacturers for antibody dilutions and concentrations were followed. It is possible that further optimization for these antibodies may allow them to perform better in the applications where they were deemed not optimal. However, as our main aim was to directly compare the performance of these two antibodies, and given at least one of the antibodies performed well for each application using our standard methods, further optimization was not performed.

Overall, based on the data from this study we would recommend the use of the Proteintech SK2 antibody for IB, as it demonstrated selectivity and sensitivity towards endogenous SK2 in the human cell lines tested. Furthermore, we recommend the ECM Biosciences SK2 antibody for IP of endogenous SK2 and for visualizing SK2 by IF methods. Furthermore, both antibodies detected non-specific proteins by IB and IF in the mouse fibroblasts used, and hence further validation will be required to determine if this is the case for other mouse cells or tissues.

## Data availability

The data referenced by this article are under copyright with the following copyright statement: Copyright: © 2017 Neubauer HA and Pitson SM

Data associated with the article are available under the terms of the Creative Commons Zero "No rights reserved" data waiver (CC0 1.0 Public domain dedication). 

**Dataset 1: Raw images of all experimental replicates for Figure 1, immunoblotting experiments.** This dataset includes uncropped blots for all experimental replicates that are represented in Figure 1. Treatments and immunoblot methods were performed as outlined in Figure 1. Blots were probed with Proteintech rabbit polyclonal anti-SK2 antibody ( **A–D**) or ECM Biosciences rabbit polyclonal anti-SK2 antibody ( **E–H**). Anti-α-tubulin antibody was used as a loading control. O/E SK2 = lysate from cells overexpressing SK2, used as a positive control to validate the correct size of SK2. Asterisks denote other protein bands that were probed using other antibodies not relevant to this study, prior to anti-α-tubulin.

DOI, 10.5256/f1000research.10336.d145416 ^18^

**Dataset 2: Raw images of all experimental replicates for Figure 2, immunoprecipitation experiments.** This dataset includes uncropped blots for all experimental replicates that are represented in Figure 2. SK2 immunoprecipitation from HEK293 cell lysate, and subsequent immunoblotting, were performed using either ( **A–C**) Proteintech rabbit anti-SK2 antibody or ( **D–F**) ECM Biosciences rabbit anti-SK2 antibody. ( **G–I**) SK2 immunoprecipitation from HEK293 cell lysates (of equal protein) treated with scrambled control siRNA (si-Neg) or SK2 siRNA (si-SK2), and subsequent immunoblotting, were performed using ECM Biosciences rabbit anti-SK2 antibody.

DOI, 10.5256/f1000research.10336.d145417 ^19^

**Dataset 3: Raw images of additional experimental replicates for Figure 3, immunofluorescence experiments.** This dataset includes additional images from experimental replicates that demonstrate reproducibility of the images presented in Figure 3. Treatments and immunofluorescence staining methods were performed as outlined in Figure 3. Images were taken at 40× magnification; scale bars = 10 µm.

DOI, 10.5256/f1000research.10336.d145418 ^20^
