# Supplementary material for: Validation of commercially available sphingosine kinase 2 antibodies for use in immunoblotting, immunoprecipitation and immunofluorescence
Source: F1000Res. 2017 Mar 23;5:2825. Originally published 2016 Dec 6. [Version 2] doi: 10.12688/f1000research.10336.2 (PMC5200942; doi:10.12688/f1000research.10336.2)
Supplement: Peer review history

# Peer review: Referee response for version 1

**DOI:** 10.5256/f1000research.11135.r18459

**Published:** 2016-12-23

**Authors:** Dagmar Meyer zu Heringdorf

**Affiliations:** 1 Institut für Allgemeine Pharmakologie und Toxikologie, Klinikum der Johann Wolfgang Goethe-Universität, Frankfurt am Main, Germany

Neubauer and Pitson have compared here two commercially available antibodies to sphingosine kinase-2 (SK2) with regard to their performance in immunoblotting, immunoprecipitation and immunofluorescence staining. As the authors point out, much less is still known about SK2 compared to SK1, and the roles of SK2 in cell death, survival, and cancer remain in large part enigmatic. Furthermore, the availability of good antibodies is certainly a matter in this area. These facts underline the importance of the author’s undertaking.

 Neubauer and Pitson present a thorough and convincing report. They selected two rabbit polyclonal antibodies raised against different regions of human SK2 with reported or predicted cross-reactivity with mouse and rat SK2. The antibodies were tested in HeLa and HEK293 cell lysates with and without siRNA knockdown of SK2, and mouse embryonic fibroblasts (MEFs) from wild type and SphK2 ^-/-^ mice. The methods are reported clearly and in exceptional detail, which is of a great value *per se*. The results show that the SK2 antibody from Proteintech performed well in immunoblotting, while the ECM antibody hardly detected SK2 but stained multiple nonspecific bands in the human and mouse cell lysates. On the other hand, the SK2 antibody from ECM, but not the Proteintech antibody, precipitated a band with the molecular weight of SK2 and caused fluorescence staining of HeLa and HEK293 cells that was sensitive to SK2 siRNA. In the MEFs, finally, neither of the two antibodies was suitable for immunofluorescence, because their staining pattern was insensitive to SK2 siRNA. The authors thus provide valuable data which can help other researchers to establish suitable protocols in their own cellular systems.

 There are only minor points which deserve a discussion. Firstly, looking at Figure 1, the SK2 bands in the Western blot appear quite faint, and the reader wonders how the immunoblots would look like when they had been exposed more strongly. However, a view into the deposited data sets suggests that compared to overexpressed SK2, endogenous SK2 is probably expressed at very low levels in HeLa and HEK293 cells. Is this indeed the fact? Secondly, the Proteintech antibody, recognizing amino acids 266-618 of human SK2a, should also recognize other isoforms of SK2 with different N-terminal lengths. Why is there only one band? Is there only one isoform of SK2 expressed in HeLa and HEK293 cells? Thirdly, a low expression of SK2 might also hamper the generation of higher quality immunofluorescence images. The ECM antibody stained nuclei and cytosolic structures in HeLa cells for which a nuclear localization of SK2 has been reported, while it caused mere cytosolic staining in HEK293 cells, similar to what others have observed with GFP-SK2. While these observations provide confidence in the specificity of the antibody, the weak staining, preventing higher resolution images, raises some doubts that a localization of endogenous SK2 at subcellular structures such as mitochondria could be detected by this antibody.

I have read this submission. I believe that I have an appropriate level of expertise to confirm that it is of an acceptable scientific standard.

# Response

**Published:** 2017-03-21

**Authors:** Heidi Neubauer

**Affiliations:** Centre for Cancer Biology, Australia

Your careful review and suggestions for improving the report are highly appreciated. In response to your comments we have included some discussion on the very low expression of SK2 protein in many cell lines which impacts on immunoblotting and immunofluorescence detection. We have also included some comments on our findings that the SK2a (SK2-S) splice isoform appears the main SK2 protein present in HeLa and HEK293 cells, despite the previous report that suggested the main SK2 mRNA in some human cell lines, including HeLa cells, encodes for the SK2b (SK2-L) splice isoform.

# Peer review: Referee response for version 1

**DOI:** 10.5256/f1000research.11135.r18381

**Published:** 2016-12-21

**Authors:** Maria Laura Allende

**Affiliations:** 1 Genetics of Development and Disease Branch, National Institute of Diabetes and Digestive and Kidney Diseases (NIDDK), National Institutes of Health (NIH), Bethesda, MD, USA

The article by Neubauer and Pitson compares two commercially available antibodies against sphingosine kinase 2 (SK2). They tested the antibodies for their performance in western blotting, immunoprecipitation and immunofluorescence using two human cell lines as well as MEFs. They concluded that the Proteintech antibody has better performance for immunoblotting while the one from ECM Biosciences works with higher sensitivity in immunoprecipitation and immunofluorescence. Both antibodies are more specific with the human cells. The results suggest that these antibodies can be used to track the level of expression of SK2. The report has detailed information on reagents utilized for each protocol as well as the experimental procedures, and it is a starting point for establishing specific optimal conditions for other investigators. The article is well written. The experiments were designed with the adequate controls. The results are presented correctly and support the conclusions.

I have read this submission. I believe that I have an appropriate level of expertise to confirm that it is of an acceptable scientific standard.

# Response

**Published:** 2017-03-21

**Authors:** Heidi Neubauer

**Affiliations:** Centre for Cancer Biology, Australia

Your positive comments and perfect understanding of the findings of this report are highly appreciated.
